# Case Management of Imported Crimean-Congo Hemorrhagic Fever, Senegal, July 2023

**DOI:** 10.3201/eid3004.231492

**Published:** 2024-04

**Authors:** Youssou Bamar Gueye, Yoro Sall, Jerlie Loko Roka, Ibra Diagne, Kalidou Djibril Sow, Alseyni Diallo, Pape Samba Dièye, Jean Pierre Diallo, Boly Diop, Omer Pasi

**Affiliations:** Ministry of Health and Social Action, Dakar, Senegal (Y.B. Gueye, Y. Sall, I. Diagne, K.D. Sow, A. Diallo, P.S. Dièye, J.P. Diallo, B. Diop);; Centers for Disease Control and Prevention, Atlanta, Georgia, USA (J. Loko Roka, O. Pasi)

**Keywords:** Crimean-Congo hemorrhagic fever, imported case, travel, vector-borne infections, viruses, zoonoses, Senegal

## Abstract

We report an imported Crimean-Congo hemorrhagic fever case in Senegal. The patient received PCR confirmation of virus infection 10 days after symptom onset. We identified 46 patient contacts in Senegal; 87.7% were healthcare professionals. Strengthening border crossing and community surveillance systems can help reduce the risks of infectious disease transmission.

Crimean-Congo hemorrhagic fever (CCHF), a severe form of hemorrhagic fever primarily transmitted to humans and animals through tick bites, is caused by CCHF virus (CCHFV). In addition, direct human contact with blood or infected tissues from viremic animals and contact with blood or secretions of an infected person have been described as transmission routes (*1*,*2*). In Senegal, the circulation of CCHFV has been reported in humans, livestock, and ticks in different areas of the country (*3*,*4*). During March–September 2023, Senegal declared a CCHF outbreak that had 8 cases distributed across 5 regions of the country (*5*). During July 2023, CCHF was diagnosed in a Senegal hospital for the 4th patient, who resided in another country. We report on the management of this imported CCHF case in Senegal.

The patient was a man in his 50s who was a trader residing in the capital of a country neighboring Senegal. He might have come into close contact with animals through his work or at home. He experienced fever, headache, and abdominal pain 2 days after returning to his rural home on July 16, 2023. The symptoms led to a consultation at a private healthcare facility in his home country, where treatment was initiated without improvement. The persistence of clinical symptoms prompted a consultation at a referral hospital in his country of residence, after which the patient’s health further deteriorated 2 days later. He had petechiae, and an abdominal ultrasound revealed hepatopathy, which prompted a family decision to seek better care in Senegal.

In Senegal, a fibroscopy and biologic tests were conducted, and results showed severe thrombocytopenia at 2,000 platelets/μL (reference range 150,000–450,000/μL). The patient was transferred to a healthcare facility that managed severe clinical cases. Because of a worsening clinical condition, including hyperglycemia and hematemesis, the patient was then transferred to the National Hospital of Pikine, Dakar, and admitted to the intensive care unit. On the 3rd day of intensive care hospitalization, the health district team collected blood samples for biologic analysis. PCR testing was positive for CCHFV 10 days after disease onset, but the patient died from multiorgan failure on the same day that PCR results were obtained.

During the case study, the investigation team identified 38 contacts in the patient’s home country; 46 contacts were identified in Senegal, most (87.7%) healthcare personnel, including doctors, nurses, and laboratory staff. No bloodborne pathogen exposure incidents were reported during patient care or while handling the patient’s samples. However, the level of infection prevention and control (IPC) was relatively low. We assessed the IPC level by using a structured assessment that had questions regarding the availability and usage of personal protective equipment, material sterilization, waste management, and the healthcare personnel’s IPC training.

We observed a delay in diagnosis for this patient despite seeking medical attention at the onset of symptoms. The time between the onset of symptoms and diagnosis was 10 days. In India, a study involving 4 CCHF cases reported an average delay of 5.75 days from symptom onset to diagnosis (*6*). However, in northern Senegal, a CCHF case was diagnosed within 3 days of symptom onset because the 4S surveillance network, a Senegal surveillance sentinel sites system (*4*), was deployed. The 4S surveillance system encompasses 25 sentinel sites distributed across the country; >1 site exists in each of the country’s 14 regions.

Healthcare personnel accounted for 87.7% of this patient’s contacts in Senegal. According to reports in the literature, healthcare professionals are one of the socioprofessional categories most affected by secondary CCHF infection (*7*,*8*). One study showed that 49% of laboratory-confirmed secondary CCHF cases were among healthcare personnel; needlestick injuries were the primary mode of exposure in 62.7% of those cases (*8*). In addition, that study identified 21 CCHF cases associated with travel (*8*). The case we report indicates that countries should adhere to Annex 1 of the World Health Organization’s International Health Regulations that defines the core capacity requirements for detecting ill travelers (*9*). During management of this CCHF case, we found no documented incidents of blood exposure, and no secondary CCHF cases were reported at the end of the 14-day contact follow-up, despite the relatively low IPC level.

In conclusion, we believe the delay in diagnosing this CCHF case resulted from the patient seeking care at multiple healthcare facilities. The healthcare personnel exposure that we identified highlights the necessity of systematically adhering to standard IPC precautions. Establishing a system for detecting potential epidemic diseases at border crossings, coupled with strengthening community surveillance, can help reduce the risks of infectious disease transmission.
